# A Probabilistic Model of Local Sequence Alignment That Simplifies Statistical Significance Estimation

**DOI:** 10.1371/journal.pcbi.1000069

**Published:** 2008-05-30

**Authors:** Sean R. Eddy

**Affiliations:** Howard Hughes Medical Institute, Janelia Farm Research Campus, Ashburn, Virginia, United States of America; Columbia University, United States of America

## Abstract

Sequence database searches require accurate estimation of the statistical significance of scores. Optimal local sequence alignment scores follow Gumbel distributions, but determining an important parameter of the distribution (*λ*) requires time-consuming computational simulation. Moreover, optimal alignment scores are less powerful than probabilistic scores that integrate over alignment uncertainty (“Forward” scores), but the expected distribution of Forward scores remains unknown. Here, I conjecture that both expected score distributions have simple, predictable forms when full probabilistic modeling methods are used. For a probabilistic model of local sequence alignment, optimal alignment bit scores (“Viterbi” scores) are Gumbel-distributed with constant *λ* = log 2, and the high scoring tail of Forward scores is exponential with the same constant *λ*. Simulation studies support these conjectures over a wide range of profile/sequence comparisons, using 9,318 profile-hidden Markov models from the Pfam database. This enables efficient and accurate determination of expectation values (E-values) for both Viterbi and Forward scores for probabilistic local alignments.

## Author Summary

Sequence database searches are a fundamental tool of molecular biology, enabling researchers to identify related sequences in other organisms, which often provides invaluable clues to the function and evolutionary history of genes. The power of database searches to detect more and more remote evolutionary relationships – essentially, to look back deeper in time – has improved steadily, with the adoption of more complex and realistic models. However, database searches require not just a realistic scoring model, but also the ability to distinguish good scores from bad ones – the ability to calculate the *statistical significance* of scores. For many models and scoring schemes, accurate statistical significance calculations have either involved expensive computational simulations, or not been feasible at all. Here, I introduce a probabilistic model of local sequence alignment that has readily predictable score statistics for position-specific profile scoring systems, and not just for traditional optimal alignment scores, but also for more powerful log-likelihood ratio scores derived in a full probabilistic inference framework. These results remove one of the main obstacles that have impeded the use of more powerful and biologically realistic statistical inference methods in sequence homology searches.

## Introduction

Sequence similarity searching was advanced by the introduction of probabilistic modeling methods, such as profile hidden Markov models (profile HMMs) and pair-HMMs [1]. When parameters are probabilities rather than arbitrary scores, they are more readily optimized by objective mathematical criteria. This enables building more complex, biologically realistic models with large numbers of parameters. For example, profile HMMs use position-specific insertion/deletion probabilities in place of the arbitrary, position-invariant gap costs of more traditional approaches such as BLAST or PSI-BLAST [2], and this allows profile HMMs to model the fact that indels occur more frequently in some parts of a protein more than others (e.g., in surface loops as opposed to buried core) [3].

More sophisticated scoring models are desirable but not sufficient. It is also necessary to be able to determine the statistical significance of a score efficiently and accurately [4],[5]. One reason that the BLAST suite of programs [2],[6] is so useful is that BLAST introduced a robust theory for evaluating the statistical significance of local alignment scores, widely known as Karlin/Altschul statistics [7]–[9]. Although the scoring technology in HMM-based profile search and profile/profile search methods is generally an improvement over BLAST and PSI-BLAST [10],[11], some problems in determining statistical significance of homology search scores have impeded the development and adoption of these or other more complex models and methods [12]. There are two main problems.

The first problem is that Karlin/Altschul statistics only rigorously apply to scores of optimal *ungapped* alignments using simple position-independent scoring systems. In this case, alignment scores follow a Gumbel distribution with slope parameter *λ* and location parameter *K* [7], and both parameters are readily calculated for any given scoring system [7],[13]. In the more relevant case of optimal *gapped* local alignments, although scores empirically still follow a Gumbel distribution for a useful range of gap costs [14], the key Gumbel *λ* parameter must be estimated by expensive computational simulation for each new scoring system [9]. Much effort aims to find better ways of determining *λ* [15]–[24]. For traditional pairwise comparison methods (e.g. BLAST), using computational simulations to determine *λ* is not a major limitation. BLAST precalculates Karlin/Altschul parameters *K* and *λ* for the small number of general scoring systems in common use [2]. However, for position-specific profile scoring models like PSI-BLAST or profile HMMs, each query specifies a customized scoring system, requiring its own *K* and *λ*. PSI-BLAST avoids using simulations to determine *λ* by restricting its profiles to fixed position-invariant gap costs, and assuming (backed by empirical results) that the *λ* of a PSI-BLAST profile is equal to the *λ* of the pairwise scoring system with the same gap costs and the most similar relative entropy (average score) per aligned residue pair [2]. For models with position-specific gap penalties, though, such as the HMMER profile HMMs used by protein domain databases like Pfam [25] and SMART [26], each model still requires a relatively expensive “calibration” by simulation before accurate E-values can be obtained. This lack of computational efficiency particularly hampers the use of profile HMMs in iterative database searches, where each iteration produces another model that needs calibration.

The second problem is that in terms of probabilistic inference, an optimal alignment score is not the score we should be calculating in a homology search. The quantity we want to calculate is the total log likelihood ratio for the target sequence(s) given an evolutionary model and a null hypothesis, *independent* of any particular alignment. The alignment is uncertain, a so-called “nuisance variable” in the inference that one wants to marginalize (integrate out). In closely related sequences, when the alignment is well determined, the optimal alignment score will approximate the total log likelihood ratio, but the more uncertain the alignment, the more the optimal alignment score and the total log-likelihood ratio differ, so remote homology detection (where alignments are most uncertain) is most affected by the approximation. Benchmarks of profile HMM sensitivity and specificity have shown that “Viterbi” scores (optimal alignment) are significantly outperformed by “Forward” scores (total log likelihood ratios, summed over all alignments) [27]. However, Karlin/Altschul statistics do not apply to Forward scores, and are not expected to [28]. The distribution that Forward scores follow had been unknown [28],[29]. Forward score distributions have been empirically fitted to various fat-tailed distributions [29], but with unsatisfactory accuracy.

Here I test two conjectures about the expected distributions of scores for full probabilistic models: that optimal gapped alignment scores (Viterbi scores) follow Gumbel distributions with a constant *λ* (just as in the ungapped alignment case) and that the expected distribution of total log likelihood ratio scores (Forward scores) asymptotes to an exponential tail with the same constant *λ*. I use simulations to show that these conjectures hold for all the models in the current Pfam database (9318 profile HMMs). In achieving these results, I modified the architecture and parameterization of profile HMMs used by HMMER [30].

## Results

This work was done as part of a reimplementation of the HMMER profile HMM software package [30] in what will become version 3 (HMMER3). For concreteness, most of the results are described in HMMER's specific context of profile HMM/sequence comparison, though I expect the same conjectures to apply more broadly (see Discussion).

### Homology Search As a Statistical Inference Problem

Let us start with a definition of Viterbi and Forward scores in terms of probabilistic inference. We have a **query** (either a single sequence or a multiple alignment), and we want to ask if a **target** sequence **x** is homologous to our query or not. To set up a hypothesis test, we specify “homology to the query” as a hypothesis (call it *H*) to be compared to (at least) one alternative hypothesis, that **x** is an unrelated sequence (call this hypothesis *R*, random). To apply probabilistic inference, both hypotheses are specified as full probabilistic models, which means that they describe probability distributions *P*(**x**|*H*) and *P*(**x**|*R*), such that 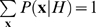 and 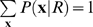 over all possible target sequences **x** = *x*_1_…*x_L_* of length *L* = 1…∞. *H* and *R* would typically be generative stochastic models such as hidden Markov models (HMMs) or stochastic context-free grammars (SCFGs) [1]. (Note that this does explicitly define a *homology search*, not merely a similarity search [31].)

Typically, model *H* will generate target residues aligned to (homologous to) residues in the query, along with deletions and insertions relative to the query, so its scoring model depends on an alignment of the query to the target. That is, model *H* directly expresses a joint probability distribution *P*(**x**,*π*|*H*), where *π* represents a particular alignment. To obtain the probability *P*(**x**|*H*), we marginalize the unknown nuisance variable *π*; that is, we sum over all possible alignments, 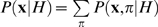.

A model might require the complete query and target sequences to be aligned and homologous – a **global** alignment model. Because biological sequences often only share homologous domains, it is more useful for *H* to permit any subsequence *i*…*j* of the query to align to any subsequence *k*…*l* of the target, while treating the remainders of the sequences as nonhomologous – this defines a **local** sequence alignment model.

The simplest random model *R* is a one-state HMM that generates sequences with each residue drawn from a background frequency distribution. This is the usual independent, identically distributed background model used when calculating standard log-odds scoring matrices, plus a geometric length distribution. In this case, there is only one possible alignment to the target sequence, and *P*(**x**|*R*) is obtained directly.

The likelihoods of *H* and *R* can be used to define at least two different log likelihood ratio scores for a target sequence **x**. The **Viterbi score** *V* is the score of the optimal alignment *π̅*:
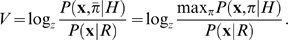



The **Forward score** *F* is obtained from the total likelihood of model *H*, a sum over all possible alignments:
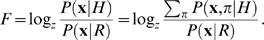



The logarithms may be taken to any base *z*. By convention, HMMER reports scores in units of bits, log base *z* = 2. Because both scores are log likelihood ratios, I will be careful to refer to Viterbi versus Forward scores, or to optimal alignment scores versus “total log likelihood ratio” scores.

The names Viterbi and Forward refer to the standard dynamic programming algorithms used to calculate these scores in the specific case of HMMs [1]. Other probabilistic models have differently named algorithms (CYK and Inside for stochastic context-free grammars for RNA analysis, for example [1],[32]), but here I will use the shorthand *V* and *F* to represent optimal alignment scores and total log likelihood ratio scores in general.

Traditional search algorithms report optimal alignment scores, so the Viterbi score is the probabilistic analog of traditional methods. However, from a probabilistic inference standpoint, the Forward score is what we want, because we are after the probability that sequence **x** is a homologue of the query – that is, the posterior probability of model *H* given data **x**, *P*(*H*|**x**) [33],[34]. The posterior is a sigmoid function of *F*:
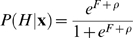
where *ρ* is a constant offset, the prior log odds ratio 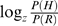.

Forward scores are not generally used in traditional sequence comparison, because they only make sense if individual alignments have probabilities *P*(**x**,*π*|*H*) that can be meaningfully summed. Forward scores cannot be calculated directly for arbitrary (nonprobabilistic) scoring systems, except by using approaches based on renormalization and partition functions, where the arbitrary scores are assumed to be unnormalized log probabilities [28], [35]–[38]).

Local optimal alignment scores of random sequences (*V* scores) are expected to follow Karlin/Altschul statistics [7],[14], a special case of a Gumbel distribution (a type I extreme value distribution) [39]:
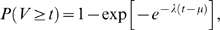
where *μ* and *λ* are location and scale parameters. Karlin/Altschul statistics give a specific dependence of *μ* on query and target sequence lengths *N* and *L*, 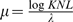, with parameter *K* essentially representing the fraction of the *NL* residue alignment lattice that is available for initiating independent local alignments. I will use the more general Gumbel notation (in terms of *μ*, *λ*) as opposed to the more usual Karlin/Altschul notation (in terms of *KNL*, *λ*) for reasons that will become clear when I consider how score distributions depend on target sequence length.

In contrast to optimal alignment scores, the distribution of Forward scores is unknown. It has appeared “fat-tailed” relative to the high-scoring exponential tail of the Gumbel distribution of Viterbi scores [28],[29].

### Expected Distributions Conjectured for Local Viterbi and Forward Scores

I made the following two conjectures about *V* and *F* scores, in the case of full probabilistic models of local sequence alignment:

- The Gumbel distribution of Viterbi scores has a fixed *λ* = log *z*, where *z* is the base of the logarithm of the log-odds scoring system.
- The high-scoring tail of Forward scores is exponentially distributed with the same *λ* = log *z*.

These conjectures are based on three main lines of argument, two of which depend heavily on the work of Bundschuh and his collaborators.

First, for Viterbi scores, Bundschuh's “central conjecture” about the distribution of optimal gapped local alignment scores states that *λ* for the Gumbel distribution is the unique positive solution of 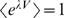 in the limit of infinite length comparisons [22],[23]. There is a strong analogy to the case of ungapped local alignments with additive pairwise residue scores *σ* *_ab_*, where *λ* is the unique positive solution of 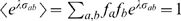 [13]. When the residue scores *σ* *_ab_* are explicitly probabilistic log-odds scores (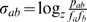 in some arbitrary logarithm base *z*) then simple algebra shows that *λ* for ungapped alignment scores is log *z*. Likewise Bundschuh's central conjecture would be satisfied by *λ* = log *z* for full probabilistic models of local alignment, when indels are included as part of the probability model rather than scored with arbitrary penalties.

Second, for Forward scores, Milosavljević proved in his “algorithmic significance” method that an upper bound for the distribution *P*(*F*>*t*) of log likelihood ratios *F* for full probabilistic models is an exponential *e* ^−*t* log z^ [40],[41]. Although this is not a tight bound, it suggests the high-scoring tail cannot be fatter than exponential, and that if it were exponential, it must have *λ*≥log *z*.

Third, for Forward scores, Yu, Bundschuh, and Hwa argued by a different approach that the high-scoring tail *P*(*F*>*t*) for scores for probabilistic sequence alignment is likely to be approximated by *e* ^−*t* log z^, i.e. again, an exponential tail with *λ* = log *z* [42]. However, they only used this result as an intermediate in a derivation showing that the scores of a new “hybrid” scoring system for local alignment would probably be Gumbel-distributed with *λ* = log *z*. They stated their approximation in the context of a full probabilistic model of global alignment, not local, and then used that result to derive a further approximation for the expected distributions of scores for a nonprobabilistic model of local alignment. However, I believe their approximation only relies on the model being fully probabilistic, not whether it is of global or local alignment.

Additionally, one expects the high-scoring tail of Forward scores to approximate the high-scoring tail of Viterbi scores (so Gumbel-distributed Viterbi scores and exponential-tailed Forward scores would have the same *λ*), because for the highest scoring sequences, the optimal alignment should contain most of the probability mass.

In practice, however, the simulation-calibrated *λ* values for bit scores of Gumbel distributions fitted to Viterbi scores of HMMER2 multihit local alignment models for 9318 Pfam 22.0 models have a mean of 0.6677, with a standard deviation of 0.051 (±8%), and a range of 0.517 to 1.337. Though the mean is suggestively close to the conjectured log2 = 0.6931, the variation is unacceptably broad, well outside traditional tolerance for useful *λ* estimates (which is typically considered to be ≤3% error [20]). Similarly, another popular profile HMM software package, SAM [3],[43], has used *λ* = log *z* in the past, but switched to simulated-calibrated *λ* values because they gave better statistical significance estimates [29]. Either something is wrong with the conjectures, or something is not quite right with profile HMMs of local alignment.

### A Generative Probabilistic Model of Local Sequence Alignment

I modified HMMER's profile HMM architecture in several details, with the main goal of achieving a uniform query entry/exit distribution in local alignments. A uniform query entry/exit distribution means that for a query profile of *N* positions 1…*N*, each choice of local alignment to a core model subsequence *i*…*j* (leaving query prefix 1…*i*−1 and suffix *j*+1…*N* unaligned) has the same probability: 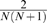, since there are 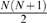 possible choices of *i*…*j*. This assumption is implicit in the traditional Smith/Waterman alignment scoring system [44], which scores identically (zero) for any choice of entry *i* and exit *j*, therefore corresponding to an implicit assumption of a uniform query fragment distribution (albeit unnormalized). HMMER's previous entry/exit distribution, in contrast, was *ad hoc* and non-uniform, causing scores to be biased by the local alignment's position in the query model. I guessed that a uniform entry/exit distribution might result in simpler, more statistically homogeneous expected score distributions that might asymptotically approach conjectured predictions faster than for nonuniform entry/exit distributions.

Besides HMMER's previous model, several other probabilistic local alignment models in the literature also imply nonuniform entry/exit distributions. For example, simple pair-HMMs for pairwise local sequence alignment imply a non-uniform (geometric) distribution over local alignment length, because they use a single residue alignment state with a self-loop and an exit probability [1]. In standard profile HMMs, I see no way to specify a uniform entry/exit distribution when delete states are present, at least not while maintaining a fully probabilistic model.

**Figure 1:**
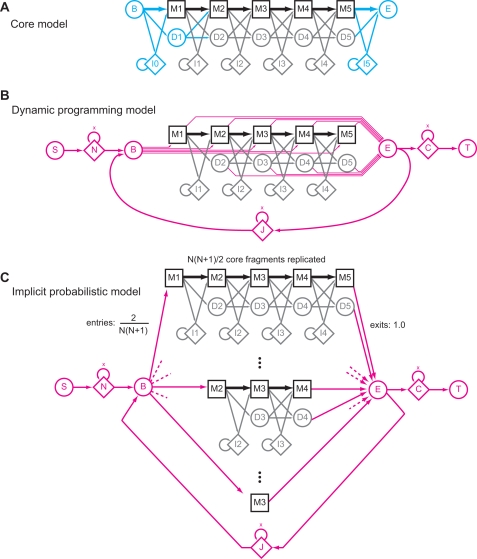
A generative probabilistic model of local alignment. (A) an example of a core model with five consensus positions. Each consensus position of the query is modeled by a *node* containing three *states*: a *match state (M)* that emits the consensus position (squares), a mute *delete state (D)* that emits nothing and deletes the consensus position (circles), and an *insert state (I)* that emits one or more residues after the consensus position (diamonds). For clarity, the emission probability distributions on match and insert states have been omitted in the figure. Nodes are numbered 1…*N* for a query of length *N* consensus positions. The three states in each node have seven transition probabilities (arrows), implementing a probabilistic model of traditional sequence alignment with affine gap penalties: the *M*→*D* and *M*→*I* probabilities correspond to gap-open costs, and *D*→*D* and *I*→*I* probabilities correspond to gap-extend costs. The core model starts and ends with mute begin (B) and end (E) states (circles). Bold arrows indicate the consensus (all match) path through the model. Blue states and transitions are either modified or removed in a configured search profile. (B) The search profile, with extra states and state transitions (magenta) enabling a model of local or glocal alignment, and unihit versus multihit alignment, as described in the text. States N, C, and J emit on transition (indicated by x's on their transition arrows), in order to be able to generate ≥0 residues rather than ≥1. (C) A partial view of the implicit probabilistic model, showing three of the possible 15 *i*…*j* query segments (1…5, 2…4, and 3) for an *N* = 5-node model, with uniform entry probabilities of 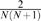 and exit probabilities of 1.0. The presence of the remaining 12 query fragments in the model is indicated by dashed entry/exit transitions and vertical ellipses.

The generative probabilistic model of local alignment that I intend to use in HMMER3 is illustrated in Figure 1.

Figure 1A shows the **core model**, which is a standard profile HMM essentially following the original formulation of Krogh *et al.* [3]. This is a model of global alignment to the original query (a multiple alignment or single sequence). The parameters in the core model (M and I residue emissions, and M, D, and I state transitions) are estimated from counts of residues and indels in the query. Details of model construction and parameter estimation in the core model follow previous work on profile HMMs, and are not particularly relevant to the results reported here except as noted.

Figure 1B shows the **search profile**, which adds extra states and state transitions to the core model to describe various kinds of alignment *modes*, including local versus glocal and unihit versus multihit. For locality with respect to a query segment, there are transitions from the begin state to any match state, and exits from any match or delete state to the end. For locality with respect to a target sequence segment, the search profile generates flanking *unannotated* segments of the target using N and C states. For a “multihit” mode, to generate multiple consistent alignments to the same query in one target sequence (either multiple domains of the same type, or separate pieces of one alignment), the model may cycle from E to the J state, generate an unannotated segment in J, and cycle back to B. The N, C, and J states are all assumed to emit residues with the same background frequencies as in null model *R*, so their log-odds emission scores are zero. This is essentially the same as the HMMER2 “Plan 7” profile architecture, but as it cannot be parameterized to achieve a uniform entry/exit distribution, the following step was taken.

Figure 1C shows the **implicit probabilistic model**. To achieve a uniform entry/exit distribution, we imagine replicating all *N*(*N*+1)/2 possible chunks *i*…*j* of the model, and assigning an entry probability of 2/*N*(*N*+1) and exit probability of 1.0 to each of these fragments. Except for these entry/exit probabilities, all other emission and transition probabilities are the same as in the search profile. Now we have a probabilistic model with a uniform entry/exit distribution, but the model is enormous. Dynamic programming on the implicit probabilistic model would be costly. A key observation is that dynamic programming on the search profile with entry probabilities set to 2/*N*(*N*+1) and exits to 1.0 is provably equivalent to doing dynamic programming on the implicit probabilistic model. Two conditions are sufficient to make this so: first, that there is a one-to-one correspondence between the sets of possible state paths in the two models, and second, that any given state path is assigned identical probability by either model. (The state transition schemes in the search profile and the implicit probabilistic model were carefully designed to fulfill these conditions.) Therefore dynamic programming on one model to find either the optimal state path or the sum over all state paths must give the same answer as the other model would. This holds so long as the probability of entering at *i* is independent of exit point *j*, which is true for a uniform entry distribution.

Therefore, the search profile is not probabilistic per se. It is a dynamic programming construct that calculates correct probabilities for the implicit probabilistic model. It uses entry probabilities of 2/*N*(*N*+1) and exit probabilities of 1.0 that are properly normalized with respect to the state diagram for the implicit probabilistic model, not the state diagram for the search profile.

The N, C, J state transitions, plus the self-loop transition in the null hypothesis HMM *R*, comprise the **target length model**, so-called because this parameterization largely controls the expected length of the target sequence. For simplicity, the target length model is expressed in terms of three parameters *p*, *q*, and *r*. *p* is the self-loop transition probability for N, C, and J, so it controls the length of unannotated segments; parameterizing these states identically corresponds to an assumption that prefixes, suffixes, and intervening unannotated regions have identical length distributions. *q* is the E→J transition probability of looping around for another pass through the core model, controlling the expected number of homologous domains per target sequence (*q* = 0 puts the model in a unihit mode, and *q*>0 is a multihit mode; I will only use *q* = 0.5 here). *r* is the self-loop transition for null model *R*'s single HMM state, controlling the length distribution generated by *R*.

How should the three target length model parameters be set? I will discuss the rationale in more detail in a later section, in the context of illustrative simulation results. For now I will just state that 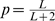, *q* = 0, and 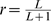 in unihit modes, and 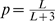, *q* = 0.5, and 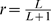 in multihit modes. That is, these model parameters are recalculated for each target, according to its length *L*: both *H* and *R* are conditional on *L*. With these choices, models *H* and *R* will both generate approximately the same mean target sequence length *L*. Previously HMMER2 used 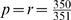 (and the same *q* = 0 or *q* = 0.5 choice of unihit versus multihit mode), independent of target sequence length. Recalculating part of the scoring system based on each target sequence's length is an unusual step, but the reason to condition the hypothesis test (both models *H* and *R*) on target length *L* will become apparent.

### Alignment “Modes”

Traditional sequence similarity search methods distinguish local, global, and glocal alignments, applying different alignment algorithms, while using the same scoring system. (A **glocal** alignment, also known as a semi-global alignment [45], is global with respect to the query 1…*N*, and local with respect to a subsequence *k*…*m* of the target; glocal alignment is useful, for example, when a profile HMM models a protein structural domain that may occur one or more times somewhere in a longer, multidomain protein sequence.) Additionally, local and glocal algorithms may allow only one aligned region per target sequence (a **unihit** alignment), or they may allow a combination of one or more aligned regions (a **multihit** alignment). The Smith/Waterman alignment algorithm [44] is a unihit algorithm, for example, whereas BLAST is multihit, implementing “sum statistics” to allow multiple consistent hits to contribute to a target's score [8].

In a probabilistic inference framework, these distinctions are not in the algorithm, but in the parameterization and architecture of the model *H*. A full (generative) probabilistic model *H* must always explicitly model the *complete* target sequence *x*_1_…*x_L_*, not just part(s) of it. This is why the HMMER model includes additional states and transitions that account for unannotated residues in the target sequence, and transitions allowing a model to loop back and generate one or more consistent alignments to the core model in the same target. Thus, an alignment *π* to a probabilistic model is always complete (and in some sense “global”) in that every residue *x_i_* in the target is assigned to a state in the model. The HMM algorithms used to score and align target sequences (Viterbi and Forward) are always the same, regardless of the configuration of the model. In HMMER, searches can be configured in any choice of local, glocal, or global combined with a choice of unihit or multihit, a total of six different standard alignment *modes*, by reparameterizing the entry/exit distribution and the target length distribution. I only explore local alignment modes in this paper, and I generally concentrate on multihit rather than unihit mode because multihit mode is more powerful.

### Local Viterbi Scores Follow Gumbel Distributions with Constant *λ*

**Figure 2:**
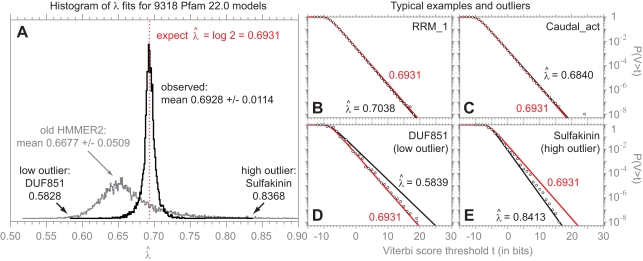
Viterbi scores follow Gumbel distributions with constant *λ*. (A) A histogram showing the distribution of 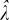 estimates determined by maximum likelihood Gumbel fits to multihit local Viterbi scores of *n* = 10^5^ i.i.d random sequences of length *L* = 400, for 9318 profile HMMs built from Pfam 22.0 seed alignments. The sharp black peak is from prototype HMMER3, with mean 0.6928 and standard deviation 0.0114, and extreme outliers indicated by arrows. The broader grey histogram is from old HMMER2, for comparison. The conjectured *λ* = log 2 is shown as a vertical dotted red line. (B,C) log survival plots (*P*(*V*>*t*) on a log scale, versus score threshold *t*) showing observed versus expected distributions for multihit local Viterbi scores for two typical Pfam models, RRM_1 and Caudal_act, for *n* = 10^8^ i.i.d. random sequences of length *L* = 400. On a log survival plot, the high-scoring tail of a Gumbel distribution is a straight line with slope −*λ*. Black circles show the observed data. The black lines show maximum likelihood fitted Gumbel distributions, with 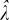 estimates as indicated. The red lines show the conjectured *λ* = log 2 Gumbel distributions, with *μ* fitted by maximum likelihood. (D,E) log survival plots for the extreme outliers DUF851 and Sulfakinin, as described in the text.

Viterbi bit scores are predicted to be Gumbel distributed with parametric *λ* = log 2. To test this prediction on many different profile HMMs, I estimated 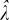
(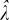 represents a maximum likelihood estimate fitted to a finite sample of scores, as distinguished from the parametric true *λ*) for 9,318 different profile HMMs built from Pfam 22.0 seed alignments, by collecting multihit local Viterbi score distributions for *n* = 10^5^ i.i.d. random sequences of length 400 generated with the same residue frequencies as the null model *R*. Figure 2 shows the results of maximum likelihood fitting these scores to Gumbel distributions. The 9,318 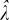 estimates are tightly clustered with mean 0.6928, consistent with the conjecture that *λ* = log 2 = 0.6931.

As examples, the top right of Figure 2 shows the score distributions for two typical Pfam models, for deep simulations with a 1000-fold larger sample size (10^8^ random sequences). As “typical” models, I chose RRM_1 and Caudal_act from Pfam 22.0. The RRM_1 model is the RNA recognition motif, a ∼72 residue domain, chosen because it is one of the Pfam domains I am most familiar with. The Caudal_act domain is the activation domain of the Caudal-like homeobox transcription factors, chosen because it is literally typical for Pfam, being closest to the median of Pfam seed alignments in three different characteristics: number of seed sequences (Pfam 22.0 median = 9; Caudal_act = 9), model length (Pfam median = 147; Caudal_act = 147), and average pairwise identity (Pfam median 36%, Caudal_act = 37%). Both observed distributions show good agreement to the predicted Gumbel of *λ* = log 2.

I examined outliers in 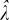 to look for models for which the conjectured *λ* = log 2 fails. If the 9318 trials were all truly Gumbel distributed with *λ* = log 2, 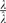 ratios (parametric over maximum likelihood estimate) should be normally distributed around a mean of 1.0 with standard deviation 0.0025 (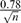, [46]), so in 9318 trials, 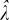 values should range from about 0.687 to 0.700 (±3.7 s.d.). The observed 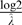 ratios do show a mean close to 1.0 (1.0008), but an s.d. of 0.0167 (six-fold higher than expected), and the 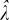
's range from 0.5828 to 0.8368. This suggests source(s) of variation beyond expected noise of fitting finite samples, and that both low and high outliers are more frequent than expected. The bottom right of Figure 2 shows multihit local Viterbi score distributions for the most extreme high and low outliers, Sulfakinin and DUF851, for deep simulations (10^8^ random *L* = 400 sequences). In both cases, a similar 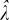 is reproduced in the second (and deeper) simulation, more evidence that these outlying values are not the result of expected statistical variation in estimation.

The low outlier DUF851 (and all other low outliers I examined) actually fits better visually to the conjectured *λ* = log 2 than to the maximum likelihood fitted 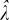. Low outliers are invariably models where the sequences in the seed alignment are highly identical. This discretizes the model's alignment scores (emission probabilities all converge to 1.0 for all consensus residues, regardless of residue type or model position) leading to a non-smooth score distribution (a stairstep-like effect is often seen, corresponding to local alignments of increasing discrete lengths 1, 2, 3…), and this stairstep gets misfit by maximum likelihood estimation. Low information content models (parameterized by entropy weighting, described later) do not show such outliers (not shown). Thus, for low outliers, the error is attributed to artifacts of maximum likelihood fitting.

The high outlier Sulfakinin (and all other high outliers I examined) does show a higher *λ* (steeper slope) than the conjectured log 2. A distinctive feature of Sulfakinin compared to other Pfam models is that it is tiny, just *N* = 9 consensus positions long. All other high outliers examined were short models. Finite-length sequence comparisons are expected to show an “edge effect” that increases the apparent *λ* relative to an asymptotic theoretical prediction, and finite-length artifacts are maximal for the shortest query models [20]. A method for compensating for “edge effect” is discussed later in the paper.

### Local Forward Scores Follow Exponential Tails with Constant *λ*

**Figure 3 pcbi-1000069-g003:**
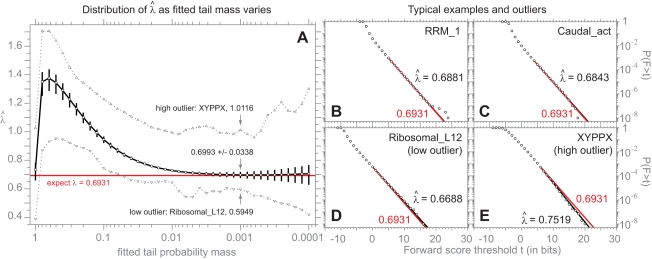
Forward scores follow exponential tails with constant *λ*. (A) a graph showing how 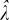 estimates for exponential distributions asymptote towards the conjectured log 2 as the fitted tail mass decreases. Each open circle is the mean of 9,318 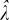 estimates, one for each Pfam 22.0 model, fitted by maximum likelihood to the high-scoring tail of multihit local Forward scores for *n* = 10^5^ i.i.d random sequences of length *L* = 400, with varying tail mass from 1.0 to 0.0001. Variation in 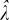 is represented by plotting quartiles (black bars) and most extreme outliers (grey triangles) in addition to the means. 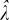 approaches the conjectured log 2 as fitted tail mass decreases, but beyond a certain point, variance increases. A tail mass of 0.001 was chosen as an appropriate tradeoff, and the mean (0.6993), standard deviation (0.0338), and outliers at that choice are annotated. (B,C) log survival plots showing observed (black circles) and expected (red lines) *P*(*F*>*t*) distributions versus score *t* for multihit local Forward scores for the “typical” RRM_1 and Caudal_act models, for *n* = 10^8^ i.i.d. random sequences of length *L* = 400. On a log survival plot, an exponential tail is a straight line of slope −*λ*. Black circles show the observed data. The black lines show maximum likelihood exponentials fitted to the 0.001 high-scoring tail, with 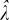 estimates as indicated. The red lines show the conjectured *λ* = log 2 exponential tails. (D,E) log survival plots for the extreme outliers Ribosomal_L12 and XYPPX.

The Forward score distribution is predicted to converge to an exponential with *λ* = log 2, with the approximation holding above some score threshold *τ*:
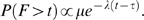



Figure 3 shows the results of maximum likelihood fitted 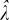 for exponential tails, for multihit local Forward scores of *n* = 500,000 i.i.d. random sequences of length 400, as a function of fitted tail mass, for 9,318 Pfam 22.0 models. We expect a tradeoff between fitted tail mass and 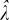 accuracy. Convergence to *λ* = log 2 is expected to occur as fitted tail mass decreases (e.g. as threshold *τ* increases), but as *τ* increases, the number of fitted samples decreases, so the accuracy of fitting 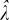 decreases. This tradeoff is seen in the data, with mean 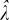 estimates closely approaching log 2 for tail masses of ≤0.001 or so. A tail mass of 0.001 was chosen as a reasonable tail mass for further characterization of Forward exponential tails.

The top right of Figure 3 shows score distributions and expected *λ* = log 2 exponential distribution of the 0.001 tail for deep (*n* = 10^8^) simulations for the “typical” RRM_1 and Caudal_act Pfam models, showing that these fits are visually satisfactory.

In this case, the survey of 9,318 models has limited power to detect significant outliers. Even with *n* = 500,000 scores, the 0.001 tail contains only 500 points, so 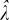 estimates will exhibit substantial stochastic variation. 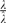 is expected to be normally distributed with mean 1.0 and standard deviation 0.045 (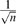, [46]), and the absolute 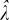 values are expected to range from about 0.590 to 0.840 (±3.7 s.d.). At the chosen tail mass of 0.001, observed 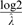 ratios have mean 0.9935 and s.d. 0.0473, with absolute 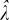 values ranging from 0.5949 to 1.0116. The variance of the 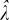 estimates is consistent with expected estimation error on the low side, but there appears to be a higher than expected frequency of large 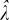 values.

The lower right of Figure 3 shows score distributions of deep simulations for the most extreme low and high outliers, Ribosomal_L12 and XYPPX, and their expected exponential tails. In both cases (and in other cases examined), deeper simulations change the 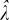 estimates, bringing them closer to log 2, suggesting expected statistical estimation error is responsible some of the discrepancies. However, for some models, including these two, 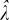 still remain significantly different from log2; Ribosomal_L12 remains −11 s.d. and XYPPX +25 s.d. away from the expected 1.0 for 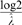 ratios for exponential tails containing 10^5^ scores.

Some low outliers exhibit the same high-identity, discretized-scores, stairstepping-distribution artifact observed with the Viterbi low outliers (DUF851 for example; not shown), but this explanation does not seem reasonable for Ribosomal_L12, where the observed score distribution appears smooth. The Ribosomal_L12 discrepancy (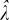 = 0.6688 differs from log 2 by 3.5%) is small and can be neglected in practice, but it is worth noting theoretically, because the Milosavljević result suggests that *λ*<log 2 should not occur. The most obvious thing that is unusual about the Ribosomal_L12 seed alignment is that it has strongly biased residue composition.

The high outlier XYPPX (and some other high outliers examined) remains a high 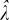 estimate in the deeper simulation (the observed 0.7519 is lower than the 0.8413 estimated in the smaller survey, but still +25 s.d. of expected given 10^5^ scores in the deeper tail). As with the Viterbi scores, XYPPX and these other high outliers are unusually small models (XYPPX is *N* = 5 consensus residues), and likely to be attributable to finite-length edge effect.

### The Target Length Model: Achieving Distributions Independent of *L*

So far, all target sequences have been a typical length of *L* = 400 residues. However, proteins range in length from a few residues to tens of thousands. One must be able to predict how the expected score distribution depends on target sequence length. For expected Gumbel distributions of traditional optimal local alignment scores, Karlin-Altschul statistics predicts that the location parameter *μ* scales as 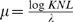 with query length *N* and target length *L*, and that the *λ* parameter (aside from finite-length edge effects) is independent of target length. That is, for each two-fold increase in target sequence length, the expected score distribution shifts by one bit.

**Figure 4:**
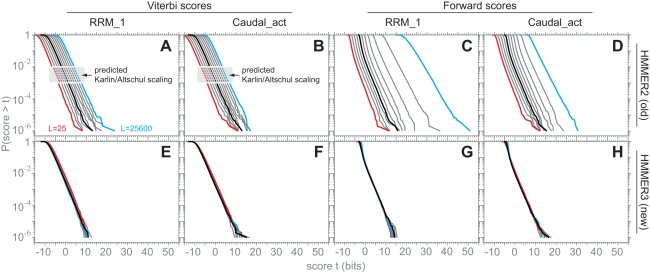
Target length modeling makes distributions length-independent. Log survival plots for multihit local Viterbi scores (left; [A,B,E,F]) and multihit local Forward scores (right; [C,D,G,H]) for the two “typical” models RRM_1 and Caudal_act, for *n* = 10^6^ i.i.d. random sequences of various lengths, for either old HMMER2 scoring (top; [A–D]) or the new target length model in prototype HMMER3 (bottom; [E–H]). Eleven target sequence lengths are used, ranging from 25 to 25,600 in steps of two-fold, with *L* = 25 shown in red, *L* = 400 shown in black, *L* = 25,600 shown in cyan, and other lengths shown in grey. Each line is the observed log survival plot, collected in 0.1 bit intervals. The grey inset in the HMMER2 Viterbi scores (A,B) shows the length dependence predicted by Karlin/Altschul statistics, with location increasing by one bit for each doubling in target sequence length. The HMMER3 results (bottom; [E–H]) show that both Viterbi and Forward scores are essentially independent of target sequence length in the new parameterization of the target length model, even for the previously problematic multihit Forward scores.

For the old target length model parameterization in HMMER2 (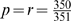 in the target length model, such that all unannotated residues assigned to N, C, J states score zero, an explicit model of Smith/Waterman's implicit assumptions), the Gumbel distributions for multihit local Viterbi scores follow the specific target length dependence predicted by Karlin-Altschul statistics, as shown in the top left of Figure 4 for two typical models. Over a range of target sequence lengths from 25 to 25,600 residues in steps of two-fold, observed score distributions are spaced in steps of one bit.

However, from a probabilistic inference standpoint, seeing the expected score increase with increasing target sequence length raises a red flag. The posterior probability *P*(*H*|**x**) should not increase as the length of a random target sequence increases. If anything, it should decrease. The more data are available (the longer the target), inference should become more accurate, and the more certain we should be that a random sequence was generated by hypothesis *R*, not hypothesis *H*.

This concern becomes a practical issue when multihit local Forward score distributions are examined for models using the HMMER2 target length model, as shown in the top right of Figure 4. These score distributions shift unpredictably, and by more than one bit per target length doubling. In absence of theory describing this length dependence, one would have to empirically determine a different exponential tail location parameter *τ* for a range of different target lengths in order to assign accurate E-values to multihit local Forward scores. Although I show later that *τ* is not hard to estimate, this is not desirable. (Unihit local Forward scores do scale by one bit per target length doubling; data not shown.)

A simple argument about the target length model appears to suffice to explain this behavior. Consider the length distribution generated by models *H* and *R*, given the length model parameters *p*, *q*, and *r*. The probability that model *R* generates a target sequence of length *L* is a geometric density:
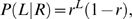
and the expected length generated by model *R* is:
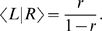



If we assume the length distribution of *H* is dominated by the N, C, J states and the target length model, and that the core model contributes negligible length (an assumption that will be most true for local alignment modes and long *L*), then the probability that model *H* generates a sequence of length *L* is a sum of Pascal distributions:
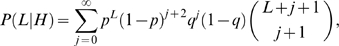
where the index *j* counts over the number of times we start a J segment. The expected length generated by model *H* can be derived from this, using the expectations for Pascal and binomial distributions:
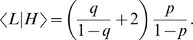



Intuitively, this follows from the fact that the expected number of times that we include a J segment is 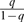. Thus, counting the two segments emitted by the N and C states, the total number of unannotated segments is 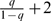, each of which follows an independent geometric distribution with expected length 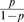.

We can then approximate the component of the log-odds Forward score that is attributable to target length modeling alone:
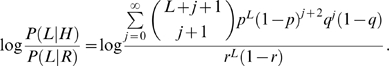
(1)


In the case of unihit modes (*q* = 0), this becomes:
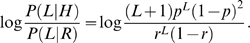
(2)So, when *p* = *r* (HMMER2's old parameterization), for unihit Forward scores, Equation 2 predicts that the target length model's score contribution will increase as log(*L*+1), essentially the same scaling for unihit local Forward scores that Karlin/Altschul statistics predicts for Viterbi (optimal alignment) scores. However, with *p* = *r*, for multihit local Forward scores, Equation 1 predicts that the length model's score contribution will scale as log(*L*+1) at small *L*, but will increase more rapidly at larger *L*. Qualitatively, this appears to be the behavior observed in Figure 4 (upper right). Intuitively, the problem is that under a target length model with *p* = *r*, model *H* favors longer sequences than model *R*, because there are at least two states (N,C) generating unannotated segments (plus additional contribution from J states in multihit mode). The longer the target sequence, the more *H* is favored, simply because it generates longer sequences with higher probability than *R*.

One way to “fix” this behavior would be to set *p* such that model *H* generates the same expected target length as model *R*. For example, in a unihit model, we might set 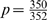, so that the N and C states each generate a mean length of 175, adding up to the same “typical protein” mean length 350 that *R* generates. But setting any constant *p* and *r* still has problems, because the length model then becomes informative - target sequences of length ∼350 get higher scores than shorter or longer sequences - and this creates a nonlinear dependence of scores on log *L*. In general we probably want target length modeling to be *uninformative*, because target sequence lengths are unpredictable. For example, the target sequence may be a fragment, or a huge multidomain protein.

How can we set an uninformative target length model? One way to do this is to make the parameterization of models *H* and *R* conditional on the length of the target sequence *L*. That is, as each new target sequence is examined, model *M* and *R* are set on the fly to generate sequences of mean length *L*:
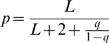


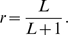



Under this scheme, according to Equation 1, the length model is predicted to contribute a nearly constant score, independent of target sequence length *L*. Empirically, using this scheme, expected score distributions indeed do become essentially target length independent (Figure 4, bottom) over a wide range of lengths *L*, both for Viterbi and for Forward scoring, and whether the model is configured for unihit or multihit alignment modes.

Target length independence is an important result. It not only means that single choices of location parameters *μ* and *τ* work for all lengths *L*; it also means that simulations that determine *μ* and *τ* can be done for a small *L*, further decreasing computational cost.

### Fast Determination of Location *τ* for Forward Tails

For the expected Gumbel distribution of local Viterbi scores, the location parameter *μ* can be determined by a maximum likelihood Gumbel fit [46] to a small simulation. When *λ* is known, *n* = 200 Viterbi scores of random sequences of *L* = 100 suffices to determine *μ* with a standard deviation of 0.1 bits. This estimation error is within tolerance. We would accept estimated E-values within about two-fold error, corresponding to an accuracy of *μ* of ±1 bit; so if we want less than one estimate in 10,000 to deviate by that much, we want a standard deviation of <0.25 or so. The time required for this simulation is essentially negligible for most purposes. For *n* = 200 sequences of length *L* = 100 and the “typical” Pfam model Caudal_act, it takes about 40 milliseconds to estimate *μ*.

It is more difficult to efficiently determine the location parameter *τ*, the base of the exponential tail of expected Forward scores. Few samples fall in the small probability mass of the tail. To obtain 200 high-scoring samples in a 0.1% exponential tail, we would still need to score 200,000 simulated random sequences, largely obviating any advantage of knowing *λ*.

After unsuccessfully exploring several alternative approaches, I adopted the following *ad hoc* method. A Gumbel distribution of *unknown* *λ* is fitted to *n* = 200 Forward scores of random sequences; the Gumbel *μ* and *λ* from this simulation are used to predict the score threshold *t* at which *P*(*F*>*t*) = 0.04 (the 4% tail); this *t* is then taken to be *τ* for the location of the base of the high-scoring 4% Forward score tail. 4% was carefully chosen. Because Forward scores are not Gumbel distributed, and appear fat-tailed with respect to a maximum likelihood fitted Gumbel of unknown *λ*, the true tail mass *P*(*F*>*t*) is systematically underestimated by a Gumbel fit. On the other hand, because the Forward survival curve approaches its exponential asymptote of *λ* = log *z* from above, if we did accurately estimate *P*(*F*>*τ*) at low score thresholds and used that to locate the base of our exponential tail, that exponential tail would overestimate (be above) the tail probability mass at higher scores. The choice of 4% was optimized by trial and error as a point at which these opposing systematic errors are well balanced; the fitted exponential tail deliberately underestimates *P*(*F*>*t*) at lower scores where the Forward distribution still appears fat-tailed, in order to become accurate in the highest-scoring tail (*P*(*F*>*t*)<0.001 or so) where the Forward distribution has converged to an exponential.

Using *n* = 200 Forward scores of random sequences of *L* = 100 suffices to determine *τ* with a standard deviation of 0.2 bits, and costs 330 msec for the “typical” Caudal_act model.

### Finite-Length “Edge Effect” on *λ*

For Karlin/Altschul statistics, the apparent *λ* for finite-length comparisons is known to increase for smaller sequences and weaker (lower relative entropy) scoring systems. Intuitively, finite length edge effect arises because the number of places that an alignment can start while still achieving a given length is less than *NL*, and achieving the highest scores requires the longest alignments (so the higher scoring alignments have fewer start points available), and weaker average scores per position require longer alignments to reach a given total score; thus higher-scoring alignments “see” a smaller search space than lower-scoring alignments, so the probability of higher-scoring alignments is lower – the tail of the distribution falls off faster – than the asymptotic *λ* predicts. Edge effect has significant impact on BLAST's statistics and substantial effort has been made to correct for it [20].

In most of the results in Figures 2–4, edge effect is not particularly apparent. However, these models have high relative entropy per position (about 1.8 bits per match state emission distribution, compared to about 0.7 bits per aligned residue pair for BLAST's default BLOSUM62 substitution scores). High relative entropy per position results from the standard multinomial estimation procedures used for parameterizing the core profile HMM [3],[47], but has been shown to compromise the sensitivity of profile HMMs [27],[43]. We have confirmed previous observations that even an *ad hoc* method to reduce the relative entropy per position (“entropy-weighting”; [43]) greatly improves search sensitivity in HMMER [27], although, puzzlingly, the same effect was not seen by PSI-BLAST's authors [48]. Empirically, on a benchmark of structural homologs [49], an optimal target relative entropy using entropy-weighting is about 0.6 bits per match state [27]. When entropy-weighted HMMER models are used, the apparent *λ*'s for both Viterbi and Forward scores deviate slightly upwards from the conjectured *λ* = log *z*. Consistent with an edge effect interpretation [20], the magnitude of this deviation is inversely proportional both to the length of the query *N* and to the average relative entropy per match state emission distribution; on the other hand, the effect does not appear to depend as strongly on the target length *L* (data not shown).

Two different approaches have been developed for correcting for edge effect. One approach is to use corrected query and target sequence lengths *N*′ = *N*−ℓ, *L*′ = *L*−ℓ, where ℓ is the expected length of an alignment [9]. Another approach is to apply a small correction to *λ*, using 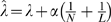, where *λ* is the true (asymptotic) value, and α is empirically determined but clearly related to the inverse of the relative entropy per position [20].

I experimented with setting an edge-corrected target length model such that the flanking nonhomology states generate *L*′ = *L*−ℓ residues for various schemes of determining an appropriate average local alignment length ℓ, but without satisfactory results. The expected alignment length length ℓ has a complicated dependence on the model, the alignment score, and the query and target lengths. In particular, my schemes tended to break down severely in the small target sequence length regime *L*≃ℓ.

Applying a correction to *λ* proved more successful. I estimate 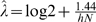, where *h* is the average relative entropy per match state emission distribution, and the 1.44 factor was empirically determined from slopes of lines fitted to *λ* versus 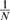 plots for models of varying *h*. Thus for typical Pfam models (*N*∼140) parameterized with standard profile HMM multinomial/Dirichlet maximum a posteriori estimation (*h*∼1.8) the correction is small (0.6931+0.0057), but for short and/or entropy-weighted models the edge effect correction has non-negligible effect.

This is only an empirically derived correction. It appears to suffice in practice, but there is clearly more going on here. A more satisfying and theoretically grounded accounting for edge effects in probabilistic local alignment is needed.

### Accuracy of E-Value Determination for Profile HMMs

In summary, the overall procedure for estimating the expected score distributions is to assume *λ* = log2, determine an edge-corrected effective lambda 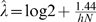 for a query model of length *N* and relative entropy per match state emission *h*, and run two small simulations (*L* = 100, *n* = 200) to determine location parameters *μ* and *τ* for the Viterbi score Gumbel distribution and the Forward score exponential tail. Because I added *ad hoc* steps (the edge effect correction and the methods for determining *μ* and *τ*) on top of the conjectures about *λ*, one now wants to know, when the complete procedure is put together, how accurate are the resulting E-values for profile HMM searches?

**Figure 5:**
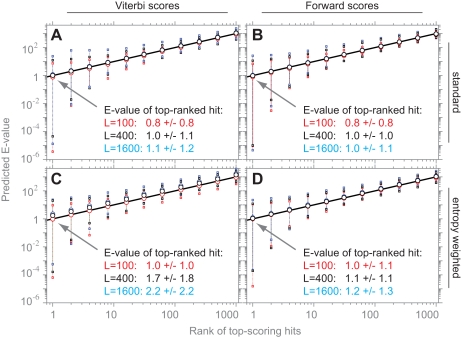
Accuracy of E-value determination. Plots of predicted E-value versus actual rank, for multihit local Viterbi scores (A,C) and multihit local Forward scores (B,D), using models with either the standard profile HMM multinomial parameterization used in the rest of the paper (A,B) or “entropy-weighted” models of reduced information content (C,D). Each plotted point (open circles) is the mean of 9,318 profile HMM searches of *n* = 10^5^ target sequences of three different target lengths: *L* = 100 (red), *L* = 400 (black), and *L* = 1,600 (cyan). The extreme outliers for each point are shown by squares and dotted vertical lines. (Interquartile ranges are smaller than the circles plotted for the means.) The expected result, of E-value equal to observed rank, is shown as a black line. Displayed text shows means and standard deviations for predicted E-values of the top-ranked score in each search, which should be (and is) about 1.0.

Figure 5 shows the results of searching 9,318 Pfam 22.0 models (either parameterized by the standard approach, or using entropy-weighting to yield lower information content models), against three different databases of 10^5^ random sequences, of lengths *L* = 100, 400, and 1600, collecting multihit local Viterbi and Forward scores, and plotting predicted E-value for the top 1000 scoring hits versus rank. If E-value estimation were perfect, we expect these points to disperse around a straight line of slope 1 (the E-value of the top hit should be 1, the E-value of the 10th ranked hit should be 10, and so on). As expected, the mean predicted E-values are indeed tightly dispersed around a straight line of slope 1. Each mean is derived from 9,318 trials, so we expect the outlying minimum E-value for the top-ranking score to be on the order of 1/9318, or about 1×10^−4^. The minimum predicted E-values for each of the six searches (Forward vs. Viterbi, three choices of length) range from 2.2×10^−4^ down to 3.7×10^−6^, basically within expectation (the 3.7×10^−6^ is significantly low, but just barely so; *P* = 0.03 to occur by chance in 9,318 trials). Some small systematic deviations from expectation can be seen on close examination, the most significant of which is in the Viterbi scores of entropy-weighted models for long (*L* = 400 and *L* = 1600) target sequences: this is where the apparent “edge effect” of low information content models is having its greatest impact.

Though statistically significant errors in E-value accuracy remain, for practical purposes they are tolerably small. Moreover, they are almost invariably in the conservative direction. That is, we would rather slightly underestimate *λ* than overestimate it. If we underestimate *λ*, we overestimate E-values and miss some true positive homologs without compromising our false positive rate. A design goal of HMMER is to accurately estimate and control false positive rates in large-scale automated analyses.

## Discussion

The most immediate benefits from this work are that for profile HMM searches, the statistical significance of both Viterbi and Forward scores can be calculated efficiently without expensive simulation. This enables substantial accelerations in the use of Viterbi scores, and more importantly, it opens the way to a broader use of more powerful Forward scores.

Although I have done the simulations in the specific context of HMMER, the local alignment model is not specific to HMMER. It is a generalized probabilistic local alignment model with a uniform entry/exit distribution. Because position-independent substitution matrix scores and gap costs are just a special case of position-specific profile scores, the same model can be used to parameterize standard Smith/Waterman local alignments [44] probabilistically. From a computational standpoint, optimal (Viterbi) local alignment for profile HMMs is essentially identical to Smith/Waterman alignment, with the same *O*(*NL*) computational complexity, and the Forward algorithm is a minor modification of Viterbi (replacing max operations with sums). Existing profile HMM implementations are two orders of magnitude slower than BLAST, but this is only because they are still using full dynamic programming (so running times are comparable to other unaccelerated Smith/Waterman implementations). There is no reason why the same heuristics that BLAST uses to accelerate Smith/Waterman cannot be applied to accelerate profile HMM searches. Similarly, existing nonprobabilistic sequence alignment methods, including BLAST, can be modified (with the addition of a few transition parameters) to accomodate the probabilistic parameterization described here.

The same conjectures are also expected to hold for local alignment scores for probability models of more than just linear sequence alignment. For example, our preliminary results indicate that local alignment scores for profile stochastic-context free grammars (SCFGs; models of RNA structure and sequence) obey the same conjectures for both CYK and Inside scores (analogous to local Viterbi and Forward scores) (DL Kolbe and SRE, unpublished results), which should help in efficiently and accurately calculating E-values for profile SCFG searches for structural RNAs [32],[50].

However, at least three important points limit any conclusions I can try to draw about how widely the conjectures might hold.

First, the same conjectures ought to hold for glocal and global alignment models. Nothing in the conjectures' rationale required the probabilistic models *H* and *R* to be configured in any particular way. However, based on previous work on glocal and global alignment scores, it is unlikely that these score distributions are going to exhibit a *λ* = log *z* simple exponential tail for biologically relevant model and sequence lengths [45],[51]. Indeed, in preliminary experiments I have observed glocal score distributions converging to *λ* = log *z* Gumbels for Viterbi scores and *e* ^−*t* log z^ exponential tails for Forward scores only for the smallest HMMs, the largest target sequences, and the most extreme tails *E*<<1. This may suggest that the conjectures hold only asymptotically, with glocal or global alignment score distributions converging slower than local score distributions.

Second, if any probabilistic local alignment model *H* should work, why would the prototype HMMER3 profile HMM architecture and parameterization be necessary to obtain these results, compared to HMMER2's local alignment scores? This again indicates that score distributions are more sensitive to details of model parameterization than the conjectures' generality would suggest. I believe the uniform local entry/exit distribution to be the important difference, again possibly because this makes score distributions reach asymptotic behaviors more quickly. However, I have not dissected the two implementations and tested specific differences one at a time, because it is not feasible to emulate HMMER2 in HMMER3's implementation (and vice versa). Moreover, perhaps inconsistent with my thinking, the other popular profile HMM software package, SAM, uses a nonprobabilistic strategy of scoring zero for local entry/exit by analogy to Smith/Waterman, which ought to produce an implicit uniform entry/exit distribution, but the SAM implementors have gone away from assuming a fixed *λ* (using Milosavljević's algorithmic significance test) and now use simulation-calibrated E-values instead [29].

Third, it is trivial to produce an example of a probabilistic model *H* that gives expected score distributions deviating strongly from the conjectures: set *H* = *R*, and all log odds ratio scores become zero (and thus *λ* = ∞). The conjectures must break down as the relative entropy between *H* and *R* approaches zero.

These issues show the main limitation of the simulation-based approach I have taken. Proper understanding of the regimes in which the conjectures break down requires a mathematical analysis, not simulations limited to a particular problem domain. Such analysis would be desirable, and it could lead in fruitful new directions. For example, the fact that HMMER3 glocal score distributions do appear to asymptote towards the conjectures (albeit not for a practical range of tail probability mass nor query and target lengths) seems promising. A general approach for estimating statistical significance of global or glocal gapped alignment scores, under traditional (arbitrary) scoring systems, largely remains elusive, despite significant effort and progress [45],[51]. Perhaps – though this is only a guess – such problems could become more amenable to mathematical analysis under the simplifying constraints imposed by a fully probabilistic scoring system. For example, the troublesome “log-linear transition” of traditional alignment scores [52] never occurs; the expected score of extending a full probabilistic alignment by an additional residue is always nonpositive.

Another problem that will need more attention is finite length effects. The finite length edge effect described for BLAST scores [20] is not the only finite length effect that can impact score distributions. Another is that there is a maximum score threshold (i.e., the score of a global, ungapped, 100% identical alignment) beyond which the probability of a higher score is just zero, so expected distributions will deviate down as they approach this maximum score threshold. In typical sequence alignments, where both the query and the target are on the order of hundreds of residues, this effect is negligible. In profile HMMs, however, where some Pfam models are quite short (as small as *N* = 5), a maximum score effect appears to be in play, especially for unihit mode models with low information content (entropy-weighted) parameters. Fortunately, any such errors will be in the conservative direction, compromising sensitivity instead of specificity (HMMER would overestimate E-values for such models).

This work was partly inspired by the work of Yu and Hwa, who described a “hybrid” (or “semi-probabilistic”) scoring method that gives Gumbel-distributed scores with *λ* = log *z* [28],[53]. Hybrid scoring essentially amounts to taking the maximum score of the cells in the Forward dynamic programming matrix. In HMMER3, I also observe Gumbel-distributed hybrid scores with *λ* = log *z* (data not shown). The three scoring systems appear to differ in their susceptibility to finite length effects that increase in low information content models. The distribution of Forward scores seems more robust than Viterbi scores (this is seen in Figures 4 and 5), and in preliminary experiments, hybrid scores appear to be even more robust (data not shown). This might account for why they turned to hybrid scores rather than standard Viterbi or Forward scores to achieve what they dubbed “universal statistics” (meaning constant *λ*).

I have taken care to distinguish Viterbi from Forward scores, and local from glocal or global alignment modes, all of which are just choices in the same full probabilistic modeling framework. Some prior work has conflated probabilistic modeling and Forward scoring, referring to Forward scores as “probabilistic alignment scores” and arguing that probabilistic alignment scores do not follow Gumbel distributions as opposed to traditional alignment scores [28], but Viterbi scores are also probabilistic. Other prior work has argued that HMMER scores do not follow expected Gumbel statistics [49], but HMMER2's default mode is multihit glocal, not local (local alignment requires a command line option). As it happens, HMMER2 does fit a left-censored Gumbel as a best-effort approximation of the glocal score distribution, and because this is known to be inaccurate, it attempts to focus the fit to achieve highest accuracy at the critical *E*∼1 region where accurate significance estimation is important; this means that HMMER2 multihit glocal (default) mode E-values are overestimated for *E*<<1, underestimated for *E*>>1, and most accurate in the *E*∼1 region, which others have observed empirically [45].

Although most homology search methods are based on local alignment, our previous internal HMMER2 benchmarks and benchmarks of other methods [45] have suggested that glocal alignment is more sensitive and specific when conserved protein domains can be defined *a priori* (as in protein domain databases like Pfam, SMART, and CDD [25],[26],[54]). On the other hand, even with predefined domain boundaries, occasional cases of conserved subdomains and truncated database sequences make it unwise to rely solely on glocal searches. For these reasons, HMMER2 has defaulted to glocal mode, and Pfam search servers report an *ad hoc* merge of glocal and local search results. We have wanted to find a way around the need to run two searches to trade off the better statistics and robustness to unusual cases of local mode versus the better average sensitivity of glocal mode. Following results of Karplus and coworkers [43], we have recently observed that much of our previously observed difference between local and glocal mode power results from local alignments being much more sensitive to the information content of the query. When we introduce parameterization methods for controlling the model's average information content per position (such as “entropy weighting” [43]), sensitivity benchmarks of HMMER local and glocal modes become comparable [27]. I am not so concerned any more that local alignment mode will be sacrificing significant search power relative to glocal mode, and I am currently planning for HMMER3 to default to local. Whether HMMER3 will implement glocal alignment mode and glocal E-value statistics remains undecided.

It is important to distinguish generative probabilistic models of local alignment from other “probabilistic” local alignment methods that apply renormalization and partition functions to interpret traditional arbitrary scores as unnormalized log-odds probabilities [28], [35]–[38]. In a generative model, *λ* is explicitly log *z*, where *z* is the base of the log used to convert probability parameters to log odds scores. In renormalization-based approaches, the original arbitrary scores and their distribution are unchanged, so determining distribution parameters like *λ* is no simpler than in BLAST or Smith/Waterman – essentially, in a renormalization approach, one must still determine the unknown implicit probabilistic basis of the arbitrary scoring system, which means determining *λ* [13].

A limitation of this work is that I have only examined scores of independent, identically distributed (i.i.d.) random sequences with a single typical amino acid composition. Real sequences often have biased residue composition, repetitive regions, and other heterogeneities that can produce spurious high-scoring aligments, requiring additional methods to compensate [29],[48],[55]. It will be necessary to confirm previous observations that the same sorts of methods apply to Forward scores, not just to optimal alignment scores [29]. Additionally, the probabilistic inference framework admits an interesting alternative, which is to develop better explicit probabilistic models of nonhomologs (hypothesis *R*), not just of homologs (hypothesis *H*).

From a purist Bayesian perspective [33],[34],[56], one might question why we need E-values and classical statistical significance tests at all. Shouldn't a posterior probability be sufficient? It would be, if model *H* were an accurate model of the sequence space of remote homologs we want to detect. However, query sequence(s) are rarely an unbiased sample from the desired space of homologs. Our model *H* usually represents a narrow clade of known query sequences, not the broader space of homologs we want to detect. Presented with a remote homolog, the model may correctly assign it a low posterior probability (it doesn't look like it belongs to the same sequence space as our query sequences), but nonetheless, it may have a higher score than one expected by chance. A purist would say that this just shows that our model is inaccurately parameterized for our problem. This is certainly true, but better parameterization requires evolutionary models that can extrapolate what remote homologs will look like, and this has proven to be a difficult problem. Most current probabilistic evolutionary models neglect important inhomogeneities in the evolutionary process, like heterotachy (rate variation between branches), and have so far proven in our hands to be insufficient in schemes for increasing profile HMM sensitivity (Alex Coventry and SRE, unpublished results). E-values and classical statistical significance testing are of immediate utility, while development of more useful probabilistic evolutionary models remains a focus for the future.

## Methods

The HMMER3 prototype source code (together with Easel, a code library that HMMER depends on) is freely available at http://selab.janelia.org/publications/#Eddy08 under the terms of the open source GNU General Public License. This source tarball includes a 00README file with detailed command-line scripts for reproducing the results in the figures. The Pfam database is freely available at http://pfam.janelia.org. The simulation results are generated by the *hmmsim* program, which takes a profile HMM as input, generates and scores *n* random i.i.d. sequences, and outputs scores, statistics, and input files for the freely available *GRACE* graph plotting program (http://plasma-gate.weizmann.ac.il/Grace/). Maximum likelihood fitting of Gumbel and exponential distributions is implemented in the *gumbel* and *exponential* modules of Easel, respectively, following methods in [46].

In HMMER3's implementation, the local entry/exit distribution is in fact not completely uniform, for the following reason. Imagine (as an extreme illustration) a profile HMM with a “consensus” match state *M_k_* that is never reached, because the (*M*,*D*)*_k_*_−1_→*M_k_* transition probabilities are zero, and imagine that this “dead” match state generates a residue that is for some reason never seen in homologs. If the local alignment model imposed a uniform entry/exit distribution, allowing an entry transition straight into the dead *M_k_* state, then local alignments can contain the impossible residue. To avoid this, HMMER *ad hoc* weights the local entry probabilities into states *M_k_* by the probability that each *M_k_* is used in sequences generated from the model. Because by default HMMER assigns consensus match states to alignment columns that contain ≥50% residues as opposed to gap characters, the usage of each match state is generally similar and high, so the effect of this weighting is normally small (less than two-fold difference between any pair of entry positions *k*).

It was necessary to implement HMMER3 dynamic programming routines as floating point calculations. In the target length model, a ratio like 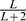 approaches 1.0 for large *L*, and roundoff/truncation error becomes an issue. The precision of HMMER2's internal scaled integer log-odds scores (in units of 0.001 bits) proved insufficient.

All computational times mentioned in the paper are measured for a single execution thread on a 3.2 GHz Intel Xeon (Dempsey) CPU, using prototype HMMER3 code compiled with the GNU C compiler (gcc) version 3.4.5 with a -O2 optimization level, running a Red Hat Enterprise Linux AS release 4 operating system.
